# Screening, Physiological Characterization, Genomic Analysis and Optimization by Conjugated Linoleic Acid Bioconversion of Two *Lactiplantibacillus plantarum* Strains

**DOI:** 10.3390/foods15172987

**Published:** 2026-08-25

**Authors:** Mengting Hu, Qingbang Li, Zhiyang Chen, Shan Lin, Pei Liu, Lin Zhou, Chuanjin Zheng, Xiaoyong Wu

**Affiliations:** 1 School of Food Science, Guangdong Pharmaceutical University, Zhongshan 528453, China; mhu53684@gmail.com (M.H.);; 2 School of Life Science and Biopharmaceutics, Guangdong Pharmaceutical University, Guangzhou 510006, China; 3 Guangdong TCM Key Laboratory for Metabolic Diseases, Guangdong Pharmaceutical University, Guangzhou 510006, China; 4 Guangdong Engineering Technology Research Center of Medicine-Food Homologous Foods, Guangdong Pharmaceutical University, Zhongshan 528453, China

**Keywords:** lactic acid bacteria screening, conjugated linoleic acid, genomic analysis, physiological characteristics

## Abstract

Conjugated linoleic acid (CLA) comprises a group of C18 fatty acids containing conjugated double bonds and has been associated with potential anti-obesity and antitumor effects. In this study, 116 presumptive lactic acid bacteria (LAB) isolates were recovered from homemade Sichuan pickles. Primary screening identified 28 CLA-producing isolates, among which strains 7# and 31# showed the highest absorbance at 233 nm (A_233_). CLA production by both strains was subsequently optimized and quantified using gas chromatography–quadrupole time-of-flight mass spectrometry (GC-Q-TOF). Under the optimized conditions, strain 31# produced 66.50 ± 3.80 μg/mL total CLA, including 52.70 ± 3.29 μg/mL c9,t11-CLA and 13.80 ± 0.51 μg/mL t10,c12-CLA. Strain 7# produced 26.79 ± 1.09 μg/mL total CLA, including 14.05 ± 0.49 μg/mL c9,t11-CLA and 12.74 ± 0.60 μg/mL t10,c12-CLA. Physiological, biochemical, and safety assessments showed that strain 31# outperformed strain 7# overall, supporting its use in further product development and mechanistic studies. Functional annotation using the COG database and pathway mapping with KEGG identified candidate genes encoding an enzyme associated with linoleic acid isomerization in both strains. Potential mechanisms underlying their different CLA-producing capacities were also examined, providing a basis for the selection and development of high-CLA-producing strains.

## 1. Introduction

Conjugated linoleic acid (CLA) refers to a class of 18-carbon fatty acids containing conjugated double bonds. It has attracted considerable attention because of its antitumor, fat-reducing, and immunomodulatory effects [1]. Traditionally, natural sources of CLA have been largely limited to meat and dairy products from ruminants [2,3,4,5,6]. CLA occurs at low levels in plant-based and low-fat foods, resulting in limited natural availability; chemical synthesis also has drawbacks, including difficulty in precisely controlling isomer composition [7]. Consequently, increasing attention has been given to the microbial production of CLA, particularly through fermentation with lactic acid bacteria (LAB). LAB are Gram-positive, non-spore-forming, facultatively anaerobic or aerotolerant bacteria that primarily convert carbohydrates into lactic acid through fermentation, and some strains exhibit probiotic properties [8]. Many LAB strains with an established history of safe use or appropriate safety evaluation are widely employed in dairy products, fermented vegetables, meat products, and plant-based beverages. However, their safety status is strain- and application-specific and should not be generalized to all LAB. In these fermented foods, acidification and the production of bacteriocins and exopolysaccharides contribute to preservation, quality, and functionality [9,10,11,12]. LAB can convert linoleic acid to CLA through linoleic acid isomerase, a capability confirmed in several species, including *Lactiplantibacillus plantarum* and *Limosilactobacillus reuteri* [2]; CLA production by LAB is highly strain-specific, with substantial variation even among *L. plantarum* strains [13,14,15,16,17]. Previous studies based on conversion efficiency have demonstrated that CLA bioconversion can be markedly affected by strain characteristics and culture conditions [13,14,15,16,17,18,19]. In parallel, studies reporting final CLA concentrations have shown that substrate selection and process optimization can substantially improve CLA production [20,21,22]. Overall, current strategies for improving microbial CLA production mainly focus on screening high-producing strains and optimizing culture conditions, cell density, and substrate delivery [18,19,20,21,22].

Although several LAB strains with high CLA-producing capacity have been reported, the CLA-producing potential of LAB originating from Sichuan pickles remains insufficiently characterized. In particular, few studies have integrated strain screening with physiological characterization, optimization of CLA bioconversion, and comparative genomic analysis to explain strain-dependent differences in CLA production. Traditional fermented vegetables, such as Sichuan pickles, provide distinctive ecological niches characterized by low pH, high salinity, and anaerobic conditions, thereby supporting diverse LAB communities [23,24]. Accordingly, Sichuan pickles represent a useful source of LAB isolates for further evaluation of probiotic-associated traits. Previous studies have also shown that some LAB isolated from naturally fermented Chinese pickles are capable of converting LA to CLA [15,25]. However, the physiological and genomic features associated with differences in CLA production among Sichuan pickle-derived strains remain poorly understood.

In this study, 116 presumptive LAB isolates were recovered from Sichuan pickles and screened for their ability to convert LA into CLA. Among the 28 isolates capable of producing CLA, strains 31# and 7# showed the highest yields and were selected for further study. Their physiological and biochemical properties were evaluated, and the culture conditions for CLA conversion were optimized, with strain 31# consistently showing better overall performance. Whole-genome sequencing was subsequently carried out to confirm the taxonomic position of both strains. COG annotation and KEGG pathway mapping were used to investigate genes involved in carbohydrate utilization, organic acid metabolism, and lipid metabolism. These analyses identified differences in metabolic potential between the two strains that may be associated with their distinct CLA-producing phenotypes. The resulting genomic information provides a basis for further functional investigation of strain-dependent CLA bioconversion and may assist in the selection of strains with favorable CLA-producing traits.

## 2. Materials and Methods

### 2.1. Materials

Ten Pickle samples were collected from household fermentation jars in Dahe Miao Township, Gusong Town, Xingwen County, Yibin, Sichuan Province, China. LA (58% purity), a CLA standard (≥99% purity), and glucose, xylose, galactose, lactose, arabinose, and fructose standards were purchased from Sigma-Aldrich (St. Louis, MO, USA). De Man, Rogosa and Sharpe (MRS) broth, MRS agar, soybean–casein digest agar, and peptone were obtained from Guangdong Huankai Microbial Science and Technology Co., Ltd. (Guangzhou, China). M17 medium and glucose-free MRS broth were purchased from Qingdao Hope Bio-Technology Co., Ltd. (Qingdao, China). Chromatographic-grade methanol and an n-hexane standard were obtained from Guangzhou Chemical Reagent Factory (Guangzhou, China). Sterile defibrinated sheep blood was purchased from Hongquan Biotechnology Co., Ltd. (Guangzhou, China). Porcine bile salts and simulated gastric, intestinal, and colonic fluids were obtained from Shanghai Yuanye Bio-Technology Co., Ltd. (Shanghai, China).

### 2.2. Instruments and Equipment

High-speed refrigerated centrifuge (Sorvall ST 16R, Thermo Fisher Scientific, Osterode am Harz, Germany); Gas chromatography–quadrupole time-of-flight mass spectrometer (8890 GC coupled with a 7250 GC/Q-TOF, Agilent Technologies, Santa Clara, CA, USA); High-shear dispersing homogenizer (T25, IKA-Werke GmbH & Co. KG, Staufen, Germany); and L5S UV–visible spectrophotometer (INESA Analytical Instrument Co., Ltd., Shanghai, China).

### 2.3. Methods

#### 2.3.1. Screening, Isolation, and Purification of LAB

Under aseptic conditions, pickle brine was collected with an inoculating loop and streaked onto MRS agar supplemented with bromocresol purple. The plates were sealed with plastic film, inverted, and incubated at 37 °C for 48 h. Colonies showing acid production, as indicated by a color change of the medium from purple to yellow, were repeatedly streaked to obtain pure cultures. The purified isolates were numbered, inoculated onto MRS agar slants, and incubated anaerobically at 37 °C for 24 h. Gram staining and microscopic examination were then performed, and Gram-positive isolates with morphological characteristics consistent with LAB were retained as presumptive LAB for subsequent identification and characterization. For short-term maintenance, the presumptive LAB isolates were stored on MRS agar slants at 4 °C. For long-term preservation, glycerol stocks were prepared and stored at −80 °C until further use.

#### 2.3.2. Assessment of CLA Production Capacity in Strains

Preparation of the LA Emulsion: An LA emulsion was prepared by mixing 1 mL of LA (58% purity, 0.903 g/mL) and 1 mL of Tween 80 with 88.4 mL of distilled water. The mixture was homogenized at 3600 rpm for 2 min to obtain a final LA concentration of 5.79 mg/mL. It was then sterilized using a 0.22 μm membrane filter and stored at 4 °C for no longer than 3 days before use.

Strain Activation and LA Fermentation: The strains described in Section 2.3.1 were subcultured twice for activation, and the second cultures were used as inocula. Each activated culture was inoculated into 9 mL of MRS broth at 3% (*v*/*v*), followed by the addition of 1 mL of LA emulsion to give a final LA concentration of 0.58 mg/mL. The cultures were then incubated anaerobically without agitation at 37 °C for 24 h.

Extraction and Primary Screening of CLA from the Fermentation Broth: Following the method described in reference [26], the fermentation broth was transferred to a 50 mL centrifuge tube and mixed with 20 mL of n-hexane. After vortexing for 10 min, the mixture was left to stand for 30 min and centrifuged at 6000 rpm and 4 °C for 10 min. The lower aqueous layer was removed, while the upper organic layer was washed twice with 20 mL of distilled water, with each wash followed by shaking and standing for 10 min. Residual moisture and the emulsion layer were removed by adding a small amount of anhydrous sodium sulfate. The organic phase was then collected in a 25 mL volumetric flask and diluted to volume with n-hexane. Absorbance at 233 nm (A_233_) was measured using a UV–Vis spectrophotometer, with unfermented MRS broth serving as the reference. Because absorbance at 233 nm is useful for the preliminary detection of conjugated double bonds but cannot specifically confirm CLA formation, this assay was used only as a preliminary screening step. Isolates with positive A_233_ values were therefore regarded as potential CLA-producing strains and were selected for subsequent chromatographic confirmation. CLA production was then confirmed by GC-Q-TOF analysis by comparing the retention times of the detected CLA methyl ester peaks with those of authentic *c*9,*t*11-CLA and *t*10,*c*12-CLA methyl ester standards.

#### 2.3.3. Optimization of CLA Production

Following the method described in reference [21], optimize CLA bioconversion conditions, inoculum size, culture medium, and lipid substrate were evaluated separately. For inoculum-size optimization, each strain was inoculated into MRS broth containing LA emulsion at 1%, 2%, 3%, 4%, or 5% (*v*/*v*) and incubated at 37 °C for 24 h. After fermentation, the lipid fraction was extracted as described in Section 2.3.2., and the absorbance at A_233_ was measured as a screening index for relative CLA formation. The inoculum size giving the highest A_233_ value was selected for subsequent experiments.

For medium screening, MRS broth, whey-based medium, and M17 broth were supplemented with the same amount of LA emulsion. Each strain was inoculated at its previously determined optimal inoculum size and cultured at 37 °C for 24 h. The fermented samples were extracted as described above, and A_233_ values were compared to identify the most suitable culture medium.

For lipid-substrate screening, LA, soybean oil, *Xanthoceras sorbifolium* oil, and a mixed oil consisting of 96.4% soybean oil, 3.0% peanut oil, and 0.6% sesame oil were tested in the selected culture medium. Each substrate was added at 0.5 mg/mL, followed by incubation at 37 °C for 24 h under the strain-specific optimal inoculum conditions. Lipids were then extracted as described in Section 2.3.2, and A_233_ was measured to select the substrate most favorable for CLA bioconversion.

#### 2.3.4. Determination of CLA by GC-Q-TOF

Fatty acids were extracted as described in Section 2.3.2, and the extract was evaporated to dryness under nitrogen. Following the method described in reference [21], acid–base methylation was performed by adding 2 mL of 0.5 mol/L sodium hydroxide in methanol, vortexing, and heating at 70 °C for 30 min. After cooling to room temperature, 2 mL of 10% hydrochloric acid in methanol was added, and the mixture was heated at 70 °C for 5 min. After cooling, fatty acid methyl esters (FAMEs) were extracted twice with 1 mL of n-hexane. GC-Q-TOF analysis was used to quantify two CLA isomers, *c*9,*t*11-CLA and *t*10,*c*12-CLA. The identities of the two isomers in the fermented samples were confirmed by matching their retention times with those of the corresponding standards analyzed under identical GC-Q-TOF conditions. FAMEs were analyzed by GC–Q–TOF using an HP-5ms column. The oven temperature was held at 60 °C for 2 min, increased to 240 °C at 5 °C/min, and held for 20 min. A 1 μL sample was injected in split mode at a split ratio of 50:1, with helium as the carrier gas. The inlet and detector temperatures were both set at 240 °C. The mass scan range was *m*/*z* 50–500, the ion-source temperature was 230 °C, and the ionization energy was 70 eV.

#### 2.3.5. Physiological and Biochemical Characteristics and Stress Tolerance of the Strains

Basic physiological and biochemical characteristics were determined according to Bergey’s Manual of Determinative Bacteriology using carbohydrate fermentation, catalase, litmus milk, and gelatin liquefaction tests [27].

Following the method described in reference [28], for growth-curve determination, activated cultures were inoculated into MRS broth at 1% (*v*/*v*) and incubated at 37 °C. Samples were collected every 2 h from 0 to 36 h, and growth was monitored by OD_600_ and viable-cell counting using serial dilution and plate enumeration. To determine the optimal growth temperature, strains were cultured in MRS broth at 20, 25, 30, 37, or 40 °C for 24 h, and growth was evaluated by OD_600_. For optimal pH determination, the initial pH of MRS broth was adjusted from 2 to 9, followed by incubation at 37 °C for 24 h.

The salt tolerance was evaluated in MRS broth containing 2–10% (*w*/*v*) NaCl under the same inoculation and incubation conditions, with growth assessed by OD_600_. For stress-tolerance assays, activated cells were harvested, washed, and resuspended, then inoculated at 3% (*v*/*v*) into simulated gastric fluid (pH 1.5 or 3.0), simulated intestinal fluid (pH 8.0), MRS broth containing 0.3% (*w*/*v*) bile salts, or MRS broth containing simulated colonic fluid. Cultures were incubated at 37 °C and sampled after 2 and 4 h, except for the gastric treatment, which was sampled after 3 h. Viable counts were determined by serial dilution and plate counting, and survival rate was calculated as (Nₜ/N_0_) × 100.

#### 2.3.6. Evaluation of the Probiotic Properties of the Strains

Following the method described in reference [29], the probiotic properties of the strains were evaluated based on their antimicrobial activity, cholesterol-removal capacity, and adhesion properties. For antimicrobial activity, strains were cultured in MRS broth at 2% (*v*/*v*) for 24–48 h at 37 °C. Cell-free supernatants (CFS) were obtained by centrifugation (8000 rpm, 10 min, 4 °C) and filtration through a 0.22 μm membrane. Indicator bacteria (approximately 10^6^ CFU/mL) were spread on agar plates, and 100 μL of CFS was added to Oxford cups. After diffusion at 4 °C for 2 h, plates were incubated at 37 °C for 12–24 h, and inhibition-zone diameters were measured. For cholesterol-removal assays, strains were inoculated at 2% (*v*/*v*) into MRS broth containing 100 μg/mL cholesterol and 0.2% (*v*/*v*) Tween 80 and cultured at 37 °C for 24 h. Residual cholesterol in the supernatant was quantified using the OPA method at 560 nm, and cholesterol removal was calculated relative to an uninoculated control. Adhesion-related properties were assessed by autoaggregation and cell-surface hydrophobicity. Cell suspensions were adjusted to OD_600_ = 0.60 ± 0.06; autoaggregation was determined after 3 and 24 h at 25 °C, whereas hydrophobicity was measured after mixing the suspension with an equal volume of xylene. Both parameters were calculated from the decrease in OD_600_ relative to the initial value.

#### 2.3.7. Identification of the Strains by 16S rRNA Gene Sequencing

Single colonies were picked from the agar slants and successively activated twice on MRS agar at 37 °C for 24 h before sequencing by Sangon Biotech Co., Ltd. (Shanghai, China). The 16S ribosomal RNA (16S rRNA) gene sequences were compared with known bacterial sequences in the GenBank database using the Basic Local Alignment Search Tool (BLAST; http://www.ncbi.nlm.nih.gov/BLAST, accessed on 26 November 2025) to identify the species with the highest sequence similarity. Representative 16S rRNA gene sequences were obtained from GenBank and aligned with the isolate sequences in MEGA version 12. A phylogenetic tree was constructed using the Neighbor-Joining method with the Kimura 2-parameter model. Both transitions and transversions were included, and uniform substitution rates across sites were assumed. The reliability of the tree topology was evaluated by bootstrap analysis with 1000 replicates. Gaps and missing data were handled using pairwise deletion.

#### 2.3.8. Whole-Genome Sequencing and Metabolic Pathway Mapping

Single colonies of strains 31# and 7#, selected for their high CLA-producing ability, were subcultured twice on MRS agar at 37 °C for 24 h. The activated cultures were then sent to Shanghai Majorbio Bio-Pharm Technology Co., Ltd. (Shanghai, China) for whole-genome sequencing. Gene functions were annotated using the KEGG and COG databases to characterize pathways associated with carbohydrate, organic acid, and lipid metabolism. Whole-genome sequencing of strains 31# and 7# was performed by Shanghai Majorbio Bio-Pharm Technology Co., Ltd. (Shanghai, China). Sequencing was conducted using a PacBio long-read sequencing platform. The final genome assemblies were generated using Flye v2.9.2. Assembly quality was evaluated based on GC-depth and k-mer frequency distributions, and genome completeness was further assessed using BUSCO v6 and CheckM v1.2.4. The average genome coverage was 66.02× for strain 31# and 70.36× for strain 7#. Protein-coding genes were predicted using Prodigal v2.6.3, and functional annotation was performed using DIAMOND v0.8.35 against the KEGG (release 20241007), eggNOG (v4.5.1), Pfam (v37), and Swiss-Prot (release 202409) databases.

#### 2.3.9. Safety Assessment of the Strains

Hemolytic activity was evaluated on TSA supplemented with 5–10% (*v*/*v*) sterile defibrinated sheep blood [30]. Activated strains were streaked or spotted onto blood agar and incubated anaerobically at 37 °C for 48 h, with *Staphylococcus aureus* and uninoculated plates serving as positive and negative controls, respectively. Hemolysis was assessed by examining the zones surrounding the colonies. Antibiotic susceptibility was determined by the disk-diffusion method [31]. Bacterial suspensions were adjusted to approximately 1.5 × 10^8^ CFU/mL, evenly spread on MRS agar, and antibiotic disks were applied after the surface had dried. Plates were incubated anaerobically at 37 °C for 24 h, and inhibition-zone diameters were measured.

### 2.4. Data Analysis

All experiments were conducted with three independent biological replicates, and each biological replicate was measured in triplicate where applicable. The results are presented as the mean ± standard deviation (SD). Statistical analyses and graphing were conducted using OriginPro 2021 (OriginLab Corporation, Northampton, MA, USA). Prior to one-way analysis of variance (ANOVA), normality was assessed using the Shapiro–Wilk test, and homogeneity of variance was evaluated using Levene’s test. When these assumptions were met, differences among groups were analyzed by one-way ANOVA followed by Tukey’s post hoc multiple-comparison test. Differences were considered statistically significant at *p* < 0.05.

## 3. Results

### 3.1. Screening of CLA-Producing LAB

**Figure 1:**
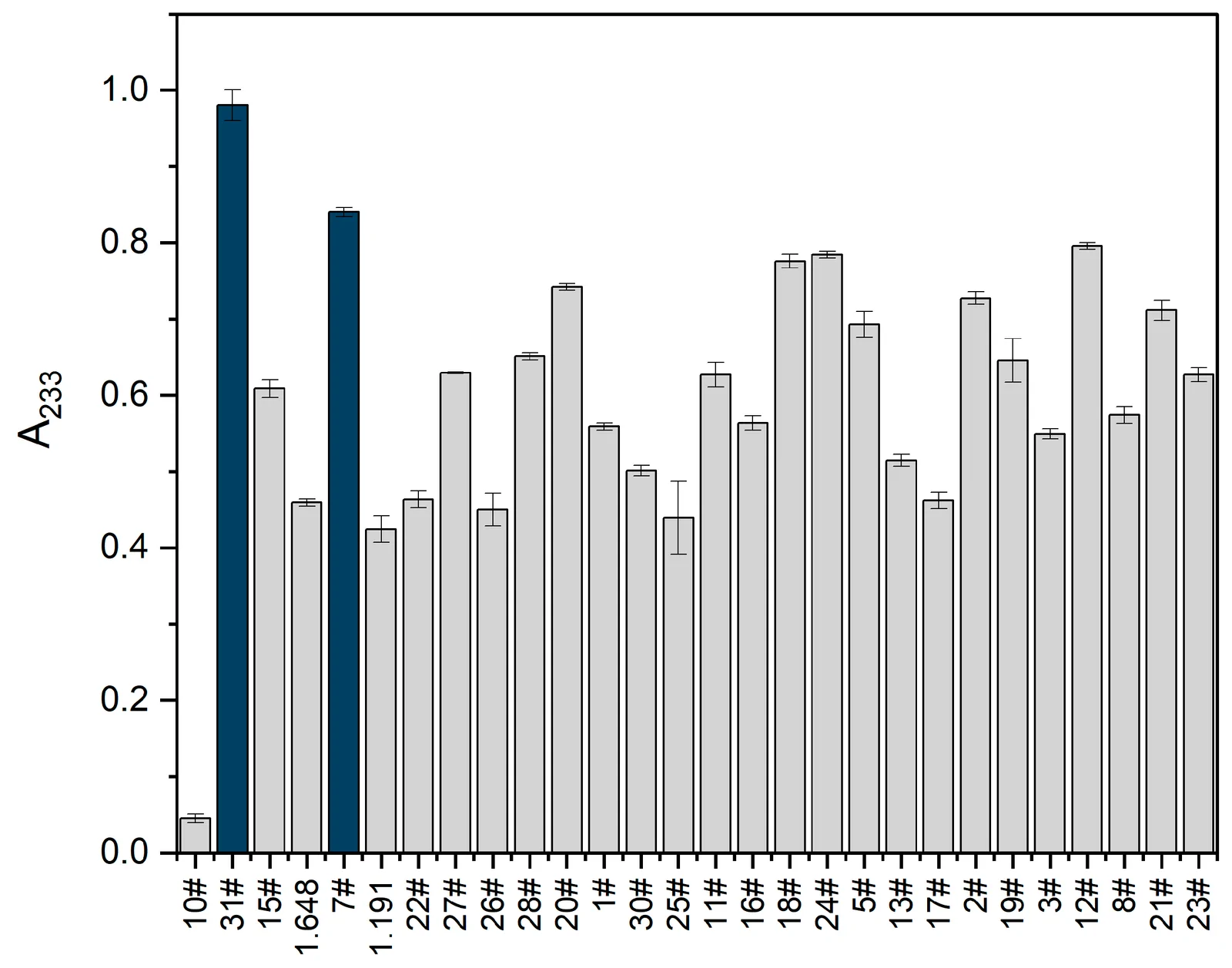
Screening Results for CLA Production. Data are presented as the mean ± SD of three independent biological replicates (*n* = 3). Blue bars indicate strains with relatively high CLA production selected during the screening, whereas gray bars indicate CLA-producing strains with relatively lower production levels.

**Figure 2:**
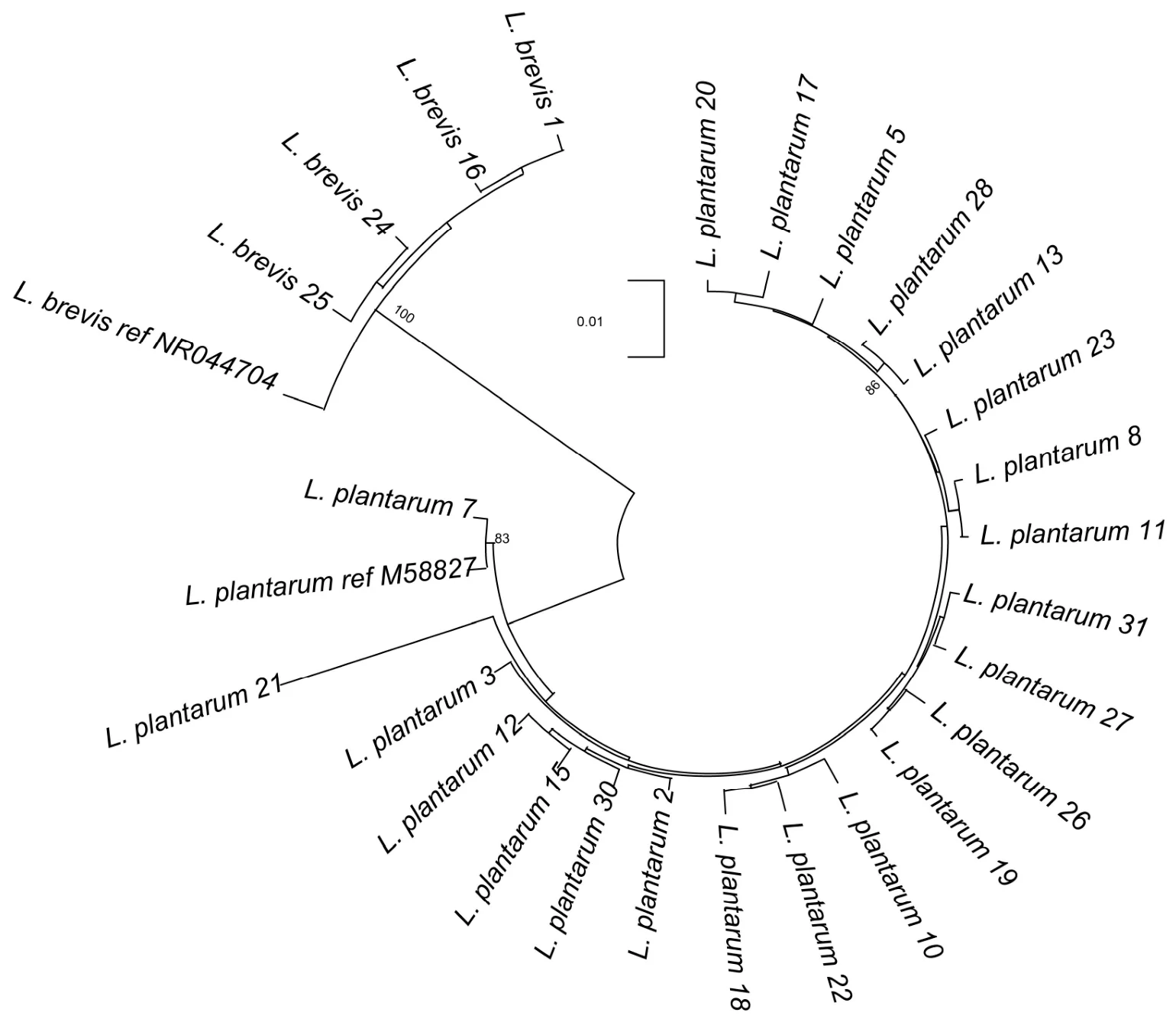
Phylogenetic tree of the LAB based on 16S rRNA gene sequences.

A total of 116 isolates producing yellow clearance zones were obtained from Sichuan pickle samples. Their colonies were milky white or yellow, circular, and regular in shape, resembling typical LAB colonies, and all isolates were Gram-positive. According to Bergey’s Manual of Determinative Bacteriology, these isolates were preliminarily identified as LAB. The CLA-producing capacities of the 116 isolates and three laboratory-preserved strains—*L. plantarum* CCFM8631, 1.648, and 1.191—were evaluated, as shown in Figure 1. Twenty-eight strains exhibited positive A_233_ values, with strains 31# and 7# showing the highest values of 0.960 and 0.926, respectively, indicating superior CLA-producing capacity. These two strains were therefore selected for further investigation. In addition, 26 LAB isolates obtained in this screening were identified by 16S rRNA gene sequencing, and the resulting phylogenetic tree is shown in Figure 2. The 16S rRNA gene sequences generated in this study were deposited in GenBank under accession numbers PZ840564–PZ840589 (Table A1).

### 3.2. Optimization of CLA Production Conditions for Two L. plantarum

**Figure 3:**
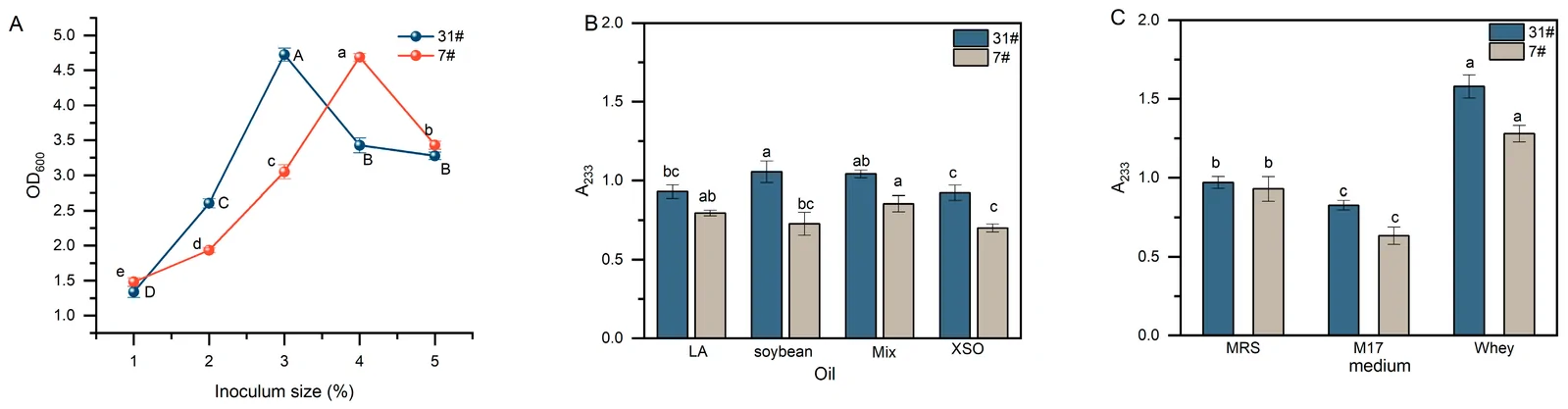
Optimization of CLA conversion conditions for the strains. (**A**) Effects of different inoculum levels on strain growth; (**B**) effects of different edible oils on CLA production; (**C**) effects of different culture media on CLA production. Data are presented as the mean ± SD of three independent biological replicates (*n* = 3). Different uppercase letters indicate significant differences among treatments for strain 31#, whereas different lowercase letters indicate significant differences among treatments for strain 7# (one-way ANOVA followed by Tukey’s post hoc test, *p* < 0.05).

Inoculum size was first optimized. The two *L. plantarum* strains were inoculated at 1–5% into MRS broth containing LA and incubated statically at 37 °C for 24 h. Optical density at 600 nm (OD_600_) was then measured, as shown in Figure 3A. For both strains, OD_600_ initially increased and then decreased with increasing inoculum size. Strain 31# reached the highest OD_600_ at a 3% inoculum (4.73 ± 0.09), significantly exceeding the other groups (*p* < 0.001). Strain 7# reached its maximum at a 4% inoculum (4.69 ± 0.06), with significant differences from the remaining groups (*p* < 0.001). An excessively high inoculum may rapidly deplete nutrients, leaving insufficient substrate for CLA conversion after exponential growth and thereby limiting CLA accumulation. Accordingly, the optimal inoculum sizes for strains 31# and 7# were 3% and 4%, respectively.

The effects of different lipid substrates on CLA production were further compared. Strains 31# and 7# were cultured for 24 h in a whey-based medium supplemented with LA, soybean oil, blended oil containing 96.4% soybean oil, 3.0% peanut oil, and 0.6% sesame oil, or *Xanthoceras sorbifolium* oil (XSO). The resulting A_233_ values are shown in Figure 3B. Significant differences were observed among the lipid substrates for both strains (*p* < 0.05). In general, cultures containing vegetable oils had higher A_233_ values than those supplemented with free LA. This may be related to the gradual release of LA from triglycerides, which could reduce the inhibitory effect of free LA on bacterial cells. The highest A_233_ value for strain 31# was obtained with soybean oil (1.08 ± 0.06), whereas blended oil gave the highest value for strain 7# (0.85 ± 0.04).

The optimal fermentation medium was subsequently selected. Strains 31# and 7# were separately inoculated into MRS, M17, and whey-based media containing 0.5 mg/mL LA and incubated statically at 37 °C for 24 h. A_233_ was then measured, as shown in Figure 3C. Medium type significantly affected the A_233_ values of both strains (*p* < 0.001). The highest values were obtained in the whey-based medium, reaching 1.581 ± 0.073 for strain 31# and 1.170 ± 0.041 for strain 7#, both significantly higher than those in MRS and M17 media (*p* < 0.05). These results indicate that the whey-based medium promoted LA-to-CLA conversion, possibly by providing suitable lactose, proteins, and low-molecular-weight nutrients. Therefore, it was selected for subsequent experiments. Strain 31# also exhibited higher A_233_ values than strain 7# with all substrates, indicating greater LA utilization and CLA conversion capacity. Therefore, the optimal conversion systems for strains 31# and 7# were whey-based medium with soybean oil and whey-based medium with blended oil, respectively.

### 3.3. Characterization of CLA Production by Two L. plantarum Strains

**Figure 4:**
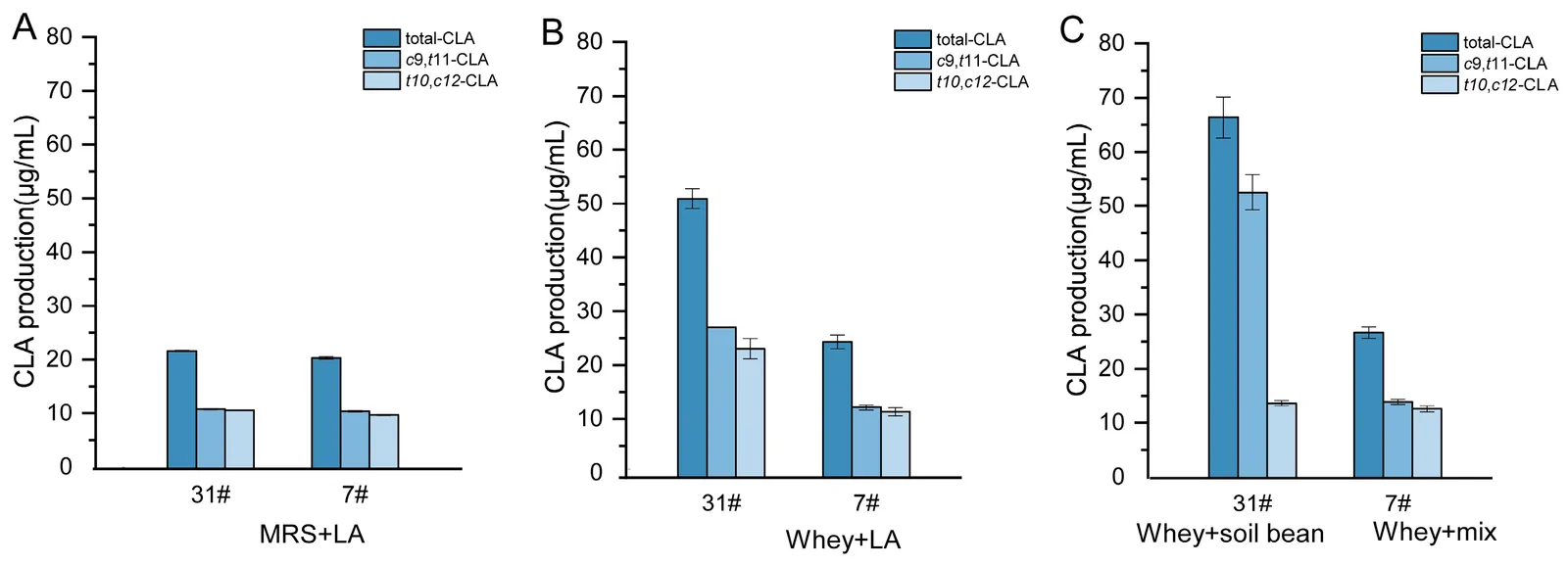
CLA production by strains 31# and 7#. (**A**) MRS medium supplemented with LA; (**B**) whey-based medium supplemented with LA; (**C**) whey-based medium supplemented with the optimal edible oil—soybean oil for strain 31# and mixed oil for strain 7#. Data are presented as the mean ± SD of three independent biological replicates (*n* = 3).

To verify the CLA-producing ability and product profiles of the two *L. plantarum* strains, CLA was analyzed using GC-Q-TOF. Figure 4 shows the CLA production of the two *L. plantarum* strains in different fermentation systems. In MRS medium containing LA, the total CLA yields of strains 31# and 7# were 21.93 ± 0.15 and 20.65 ± 0.25 μg/mL, respectively, with no significant difference. When MRS medium was replaced with whey-based medium, CLA production increased in both strains, reaching 51.81 ± 1.88 μg/mL for strain 31# but only 25.19 ± 1.25 μg/mL for strain 7#. These findings suggest that strain 31# was more efficient in utilizing free LA for CLA synthesis. Among the lipid substrates tested, soybean oil supported the greatest total CLA production by strain 31# in the whey-based medium, yielding 66.50 ± 3.80 μg/mL. Of this amount, 52.70 ± 3.29 μg/mL was c9,t11-CLA, indicating that the increase in total CLA mainly resulted from greater formation of this isomer. For strain 7#, the mixed-oil medium gave the highest total CLA yield (26.79 ± 1.09 μg/mL), but this value was only marginally higher than that obtained with free LA. Oil supplementation therefore provided little improvement in CLA production by strain 7#.

Overall, strain 31# consistently produced more CLA than strain 7# across the optimized systems and performed best with soybean oil. Therefore, strain 31# was selected as the preferred strain for subsequent CLA-enriched fermentation, with whey-based medium supplemented with soybean oil as its optimal conversion system. The relatively suitable system for strain 7# was whey-based medium supplemented with mixed oil. Plant oils may not only provide LA but also improve CLA conversion efficiency by gradually releasing the substrate and reducing the inhibitory effects of free LA on bacterial cells.

### 3.4. Physiological and Biochemical Characteristics of Two L. plantarum Strains

The carbohydrate fermentation results of the two strains are shown in Table 1. Strain 31# utilized glucose, lactose, galactose, and fructose, whereas strain 7# utilized glucose, lactose, and fructose but not galactose. As shown in Table 2, both strains decolorized and coagulated litmus milk, indicating acid production. Neither strain liquefied gelatin or exhibited catalase activity, and both were negative in the indole and methyl red tests and were nonmotile. According to Bergey’s Manual of Determinative Bacteriology, strains 31# and 7# were assigned to the genus Lactobacillus [27,32].

**Figure 5:**
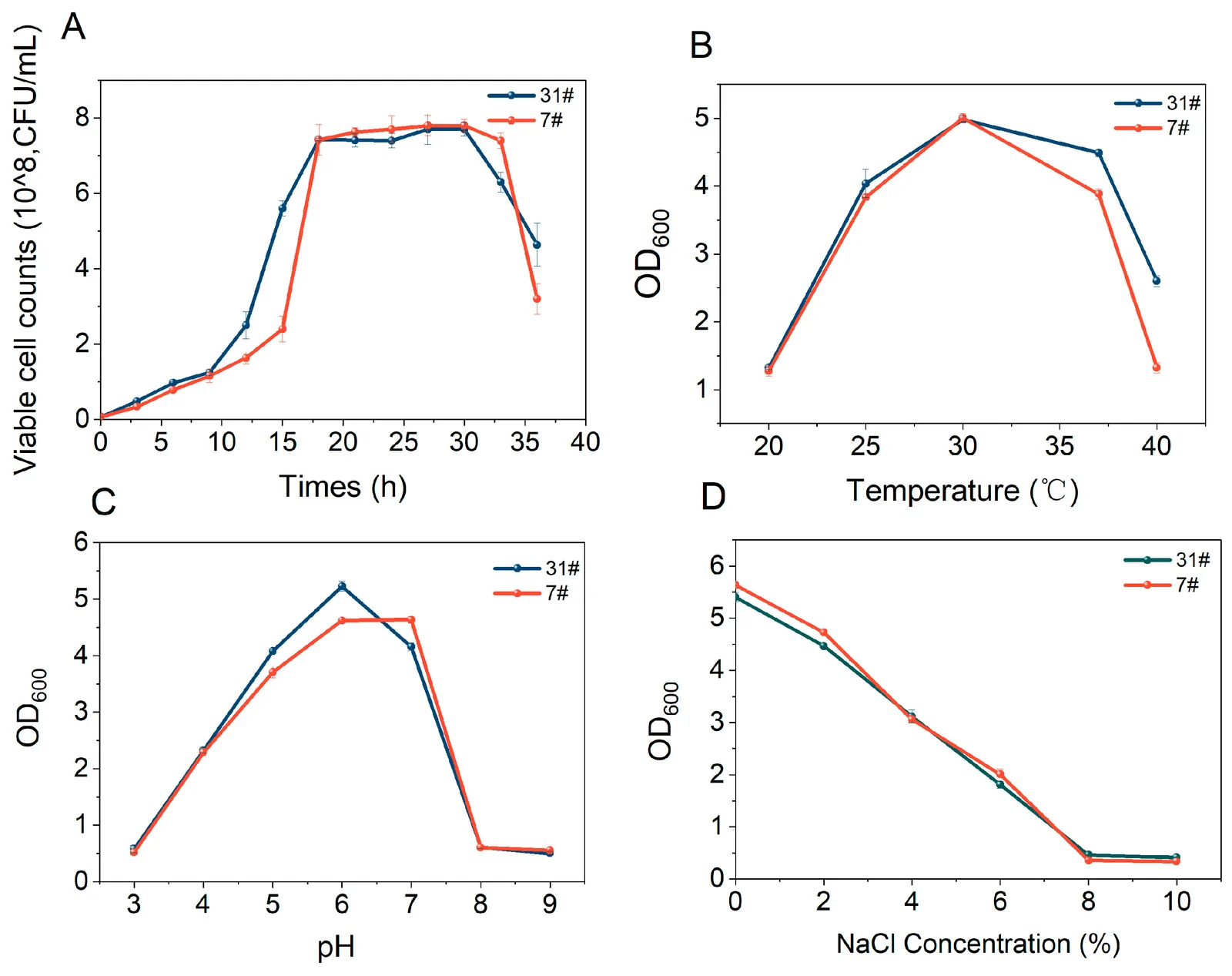
Physiological characteristics of the strains. (**A**) Changes in the viable cell counts of the two strains at different incubation times; (**B**) effects of different incubation temperatures on strain growth; (**C**) effects of different initial pH values on strain growth; and (**D**) effects of different NaCl concentrations on strain growth. Data are presented as the mean ± SD of three independent biological replicates (*n* = 3).

The growth curves of the two *L. plantarum* strains are shown in Figure 5A. Both strains exhibited an exponential phase from 9 to 18 h, followed by stationary phases of 18–30 h for strain 31# and 18–33 h for strain 7#, before entering the decline phase.

The growth of the two *L. plantarum* strains at different temperatures is shown in Figure 5B. Both strains showed the highest growth at 30 °C among the temperatures tested. At the higher temperatures tested, the decrease in OD was more pronounced for strain 7# than for strain 31#.

Both strains showed their highest growth at pH 6 (Figure 5C). OD of strain 31# varied considerably across pH 5–7, whereas that of strain 7# changed less between pH 6 and 7. These results describe differences in growth responses to pH under the conditions tested.

Strains 31# and 7# were inoculated at 1% into MRS medium containing 2–10% NaCl and cultured at pH 6 and 30 °C for 24 h (Figure 5D). Both strains showed similar decreases in OD with increasing NaCl concentrations, while strain 7# grew well at 2% NaCl.

### 3.5. Stress Tolerance of Two L. plantarum Strains

**Figure 6:**
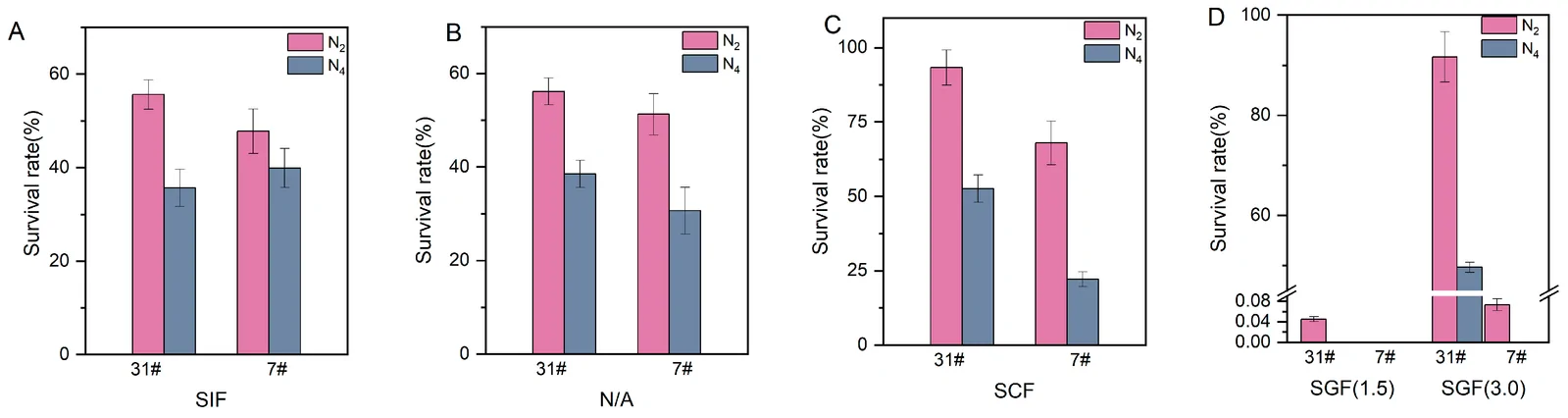
Stress tolerance of the strains. (**A**) Simulated intestinal fluid (SIF); (**B**) bile salt conditions; (**C**) simulated colonic fluid (SCF); and (**D**) simulated gastric fluid (SGF) at pH 1.5 and 3.0. N_2_ and N_4_ represent the survival rates after 2 and 4 h of treatment, respectively. Data are presented as the mean ± standard deviation. Data are presented as the mean ± SD of three independent biological replicates (*n* = 3).

The stress tolerance of the two *L. plantarum* strains is shown in Figure 6. Figure 6A–C show that strains 31# and 7# maintained survival rates above 50% after 2 h in simulated intestinal fluid, bile salt solution, and simulated colonic fluid. However, the survival of strain 7# declined markedly after 4 h in bile salt solution and simulated colonic fluid, showing a marked loss of viability with prolonged exposure. As shown in Figure 6D, both strains survived poorly in simulated gastric fluid at pH 1.5; only strain 31# remained detectable after 2 h, with a survival rate of approximately 0.04%. At pH 3.0, strain 31# showed survival rates of 91.64 ± 5.01% after 2 h and 49.62 ± 1.02% after 4 h, whereas strain 7# showed only approximately 0.08% survival after 2 h. These results indicate that strain 31# survived better than strain 7# under several of the simulated gastrointestinal conditions tested, particularly at pH 3.0. However, the extremely low survival of both strains at pH 1.5 and the poor survival of strain 7# at pH 3.0 indicate limited acid resistance, which should be considered when evaluating their probiotic potential.

### 3.6. Safety Assessment of Two L. plantarum Strains

To assess their safety for further application, the isolated strains were subjected to hemolysis testing using *Staphylococcus aureus* as the positive control. The positive control exhibited β-hemolysis, whereas both *L. plantarum* strains showed γ-hemolysis, indicating no detectable hemolytic activity under the conditions tested.

Antibiotic susceptibility of strains 31# and 7# was evaluated using previously reported interpretive criteria [31], and the results are shown in Table 3. Both strains were resistant to ciprofloxacin and trimethoprim-sulfamethoxazole. Reduced susceptibility to these antibiotics has been reported in lactobacilli and may reflect intrinsic characteristics rather than acquired resistance [33,34]. Strains 31# and 7# were resistant and intermediately susceptible to gentamicin, respectively. Reduced susceptibility to aminoglycosides may also be associated with intrinsic features of lactobacilli. Strain 31# showed intermediate susceptibility to penicillin, whereas strain 7# was susceptible. Both strains were sensitive to ampicillin, ceftriaxone, erythromycin, lincomycin, chloramphenicol, and tetracycline. Taken together, the antibiotic susceptibility profiles did not raise major safety concerns under the EFSA evaluation framework, supporting the potential use of these strains in further applications [35].

### 3.7. Evaluation of Probiotic Properties

**Figure 7:**
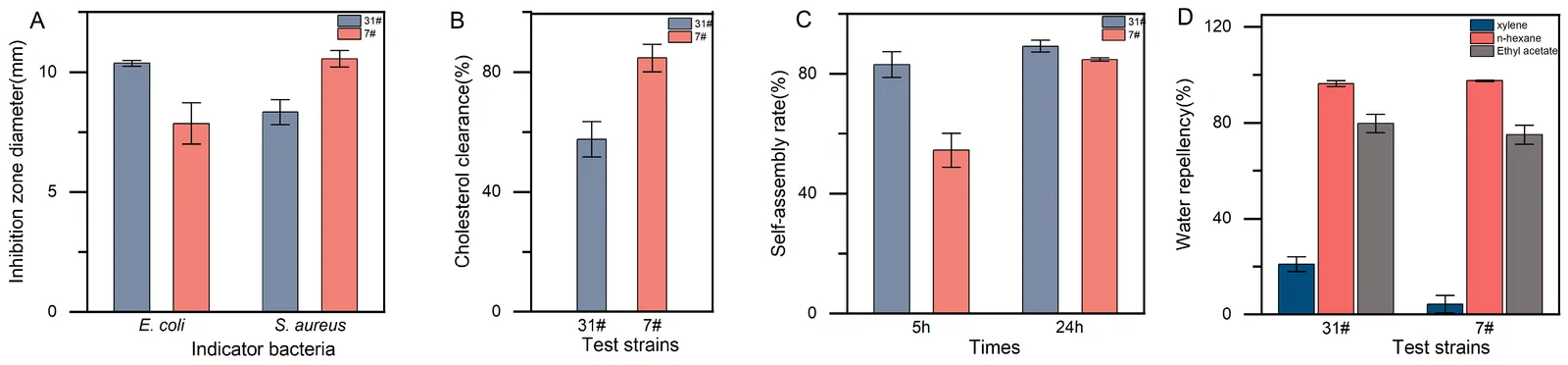
Evaluation of probiotic properties. (**A**) Diameters of inhibition zones against *Escherichia coli* and *Staphylococcus aureus*; (**B**) cholesterol removal rate; (**C**) auto-aggregation rates after 5 and 24 h of incubation; and (**D**) cell surface hydrophobicity of the strains in xylene, n-hexane, and ethyl acetate. Data are presented as the mean ± SD of three independent biological replicates (*n* = 3).

The antimicrobial activities of the two *L. plantarum* strains are presented in Figure 7A. Their inhibitory effects varied according to the indicator microorganism. Strain 31# was more effective against *Escherichia coli*, producing an inhibition zone larger than 10 mm, while strain 7# showed greater activity against *Staphylococcus aureus*. This strain-dependent activity may be related to differences in antimicrobial metabolites. Organic acids and bacteriocin-like substances are possible contributors.

The cholesterol removal results are shown in Figure 7B. Strain 7# removed approximately 84% of the cholesterol from the medium, significantly exceeding the value observed for strain 31# (approximately 58%). These results indicate a greater in vitro cholesterol-removal capacity of strain 7#. However, cholesterol removal under the present in vitro conditions should not be interpreted as evidence of a cholesterol-lowering effect in vivo.

The results are shown in Figure 7C. The autoaggregation rates of both *L. plantarum* strains increased over time. After 5 h, strain 31# exhibited an autoaggregation rate above 80%, significantly higher than the approximately 55% observed for strain 7#. After 24 h, both strains exceeded 80%, with strain 31# approaching 90%. These findings indicate strong autoaggregation, particularly for strain 31# at the earlier time point. Autoaggregation may represent a surface characteristic associated with microbial adhesion, but it does not directly demonstrate adhesion to intestinal epithelial cells or intestinal colonization.

The results are shown in Figure 7D. Both *L. plantarum* strains exhibited greater than 95% affinity for n-hexane, indicating high cell surface hydrophobicity. Strain 31# showed slightly greater affinity for ethyl acetate and xylene than strain 7#, indicating differences in cell-surface hydrophobicity between the strains. Together with the autoaggregation results, these findings describe surface properties that may be associated with adhesion-related characteristics; however, they do not provide direct evidence of adhesion to intestinal epithelial cells.

### 3.8. Genomic Characterization of the Strains

#### 3.8.1. General Genomic Features

As shown in Table 4, strains 31# and 7# had genome sizes of approximately 3.26 Mb and identical GC contents of 44.54%. Their numbers of coding sequences (CDSs), ribosomal RNAs (rRNAs), and transfer RNAs (tRNAs) were similar, indicating broadly similar general genomic features between the two strains. Both genomes contained clustered regularly interspaced short palindromic repeats (CRISPR), prophages, genomic islands, and insertion sequences. These elements were identified as structural features of the two genomes, but their presence alone does not establish specific functional or adaptive characteristics. Strain 7# contained slightly more genomic islands and insertion sequences than strain 31#, although the biological significance of this difference requires further investigation. The genome sequence data were deposited in NCBI GenBank under BioProject accession PRJNA1513201. The BioSample accession numbers are SAMN62436600 for strain 31# and SAMN62436601 for strain 7#, respectively.

#### 3.8.2. COG Functional Classification

**Figure 8:**
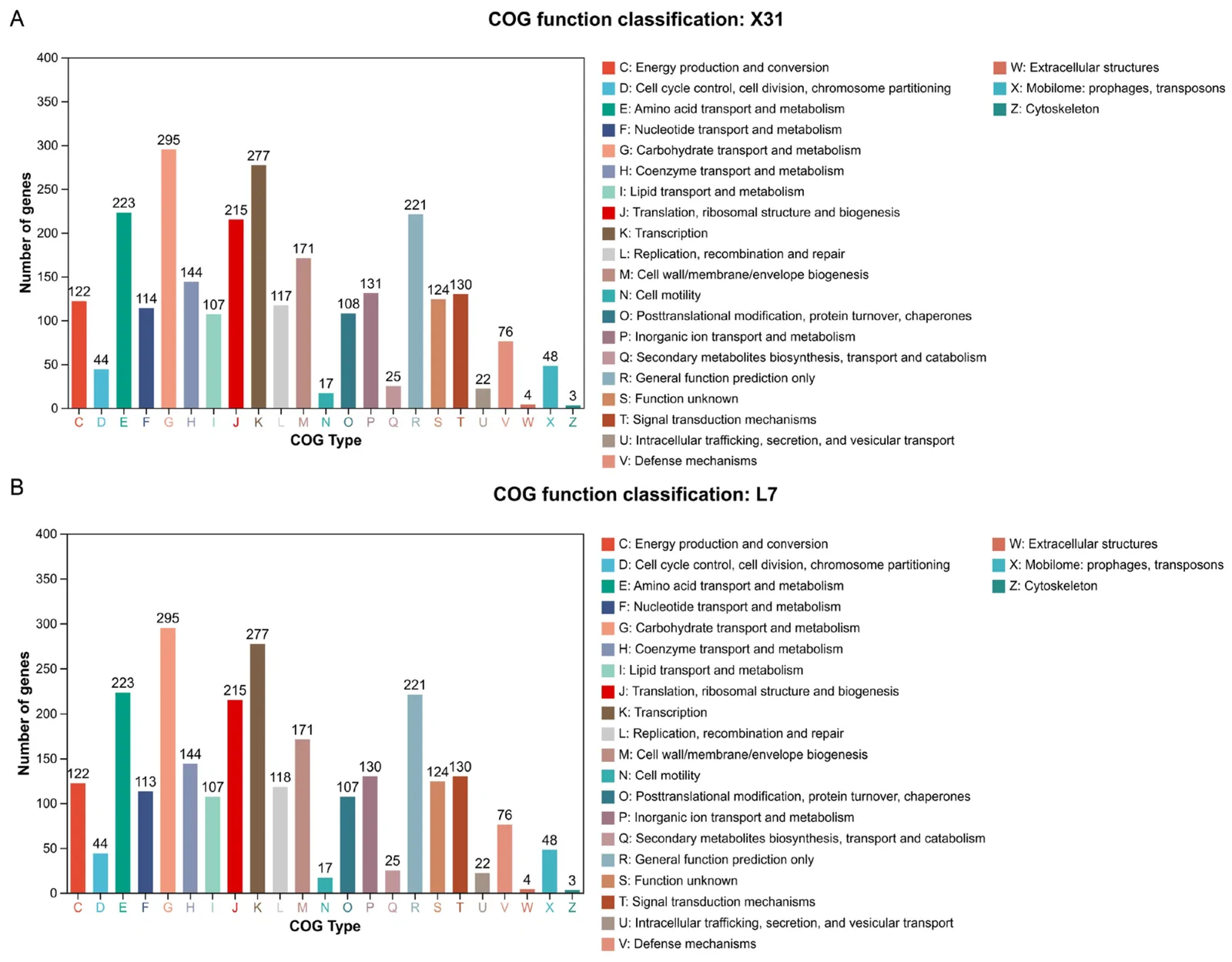
Clusters of Orthologous Groups (COG) functional classification of the strains: (**A**) strain 31#; (**B**) strain 7#.

The predicted proteins were searched against the Clusters of Orthologous Groups (COG) database using DIAMOND, with an E-value cutoff of 1 × 10^−5^. The annotated genes were distributed among 23 functional categories (Figure 8), and the overall profiles were similar between the two strains. Categories related to carbohydrate transport and metabolism (G), amino acid transport and metabolism (E), transcription (K), and translation (J) were particularly well represented, reflecting their broad metabolic capacity and conserved cellular functions. In addition, 107 genes in each strain were assigned to lipid transport and metabolism (I). Because this COG category encompasses a broad range of lipid-related functions, the number of genes assigned to it cannot be directly linked to CLA-producing capacity. The higher CLA yield observed for strain 31# may involve differences in specific gene content, gene regulation, or metabolic activity; however, these possibilities were not directly examined in this study and require further investigation.

### 3.9. KEGG Pathway Mapping

#### 3.9.1. Carbohydrate Metabolic Pathways

**Figure 9:**
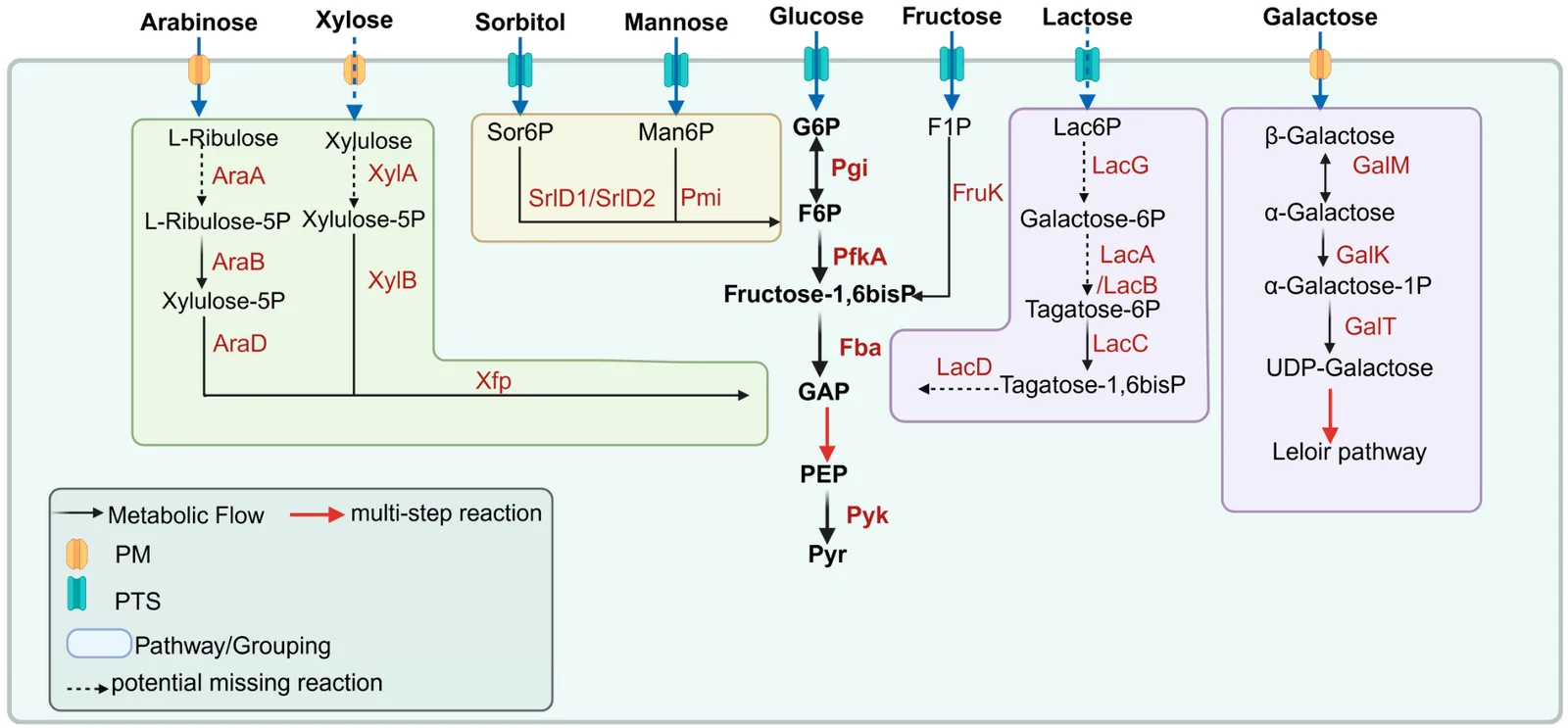
Predicted carbohydrate metabolism pathways.

The predicted carbohydrate metabolism pathways of strains 31# and 7# are summarized in Figure 9. Strain 31# contained genes associated with the transport and utilization of fructose, mannose, sorbitol, glucose, lactose, and galactose. GalK, GalM, and GalU were identified in the galactose metabolism pathway. Strain 7# showed a generally similar carbohydrate metabolism profile; however, the step linking galactose 1-phosphate to UDP-galactose was incomplete. Although the lactose-specific transport system appeared incomplete in both genomes, both strains fermented lactose in the phenotypic assay, and the underlying uptake mechanism was not resolved in this study.

#### 3.9.2. Metabolic Pathways of Organic Acid Production

**Figure 10:**
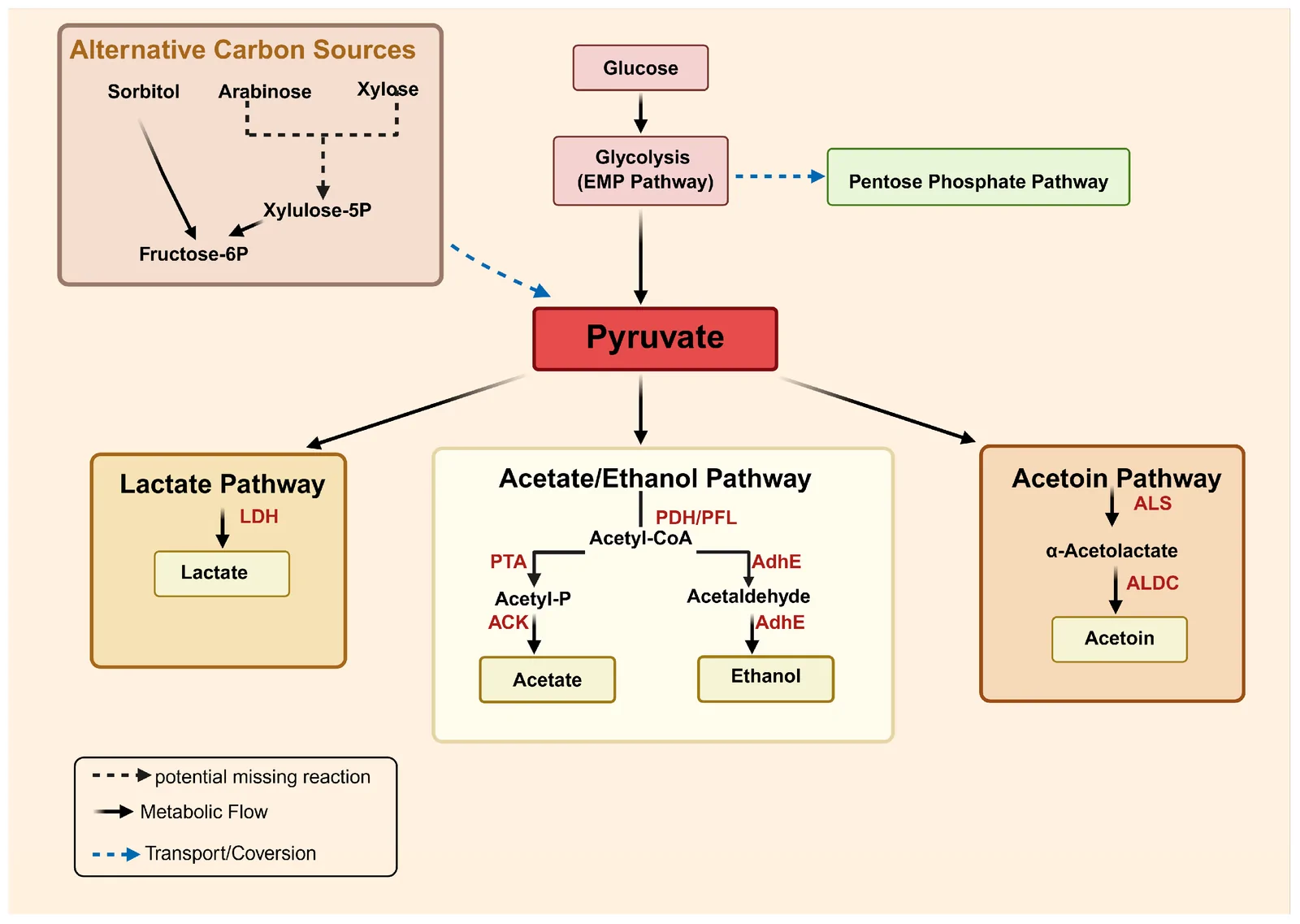
Predicted metabolic pathways of organic acid production.

The predicted pathways involved in organic acid metabolism are shown in Figure 10. Both strains contained lactate dehydrogenase (LDH; EC 1.1.1.27), enzymes associated with acetoin formation, and phosphoketolase (Xfp). Genes involved in arabinose and xylose uptake were incomplete in both genomes, and genes required for malolactic fermentation were not clearly identified. A notable difference was observed in the acetate-forming branch: strain 31# contained both phosphotransacetylase (PTA) and acetate kinase (ACK), whereas the corresponding pathway was incomplete in strain 7#.

#### 3.9.3. Lipid Metabolism Pathways

**Figure 11:**
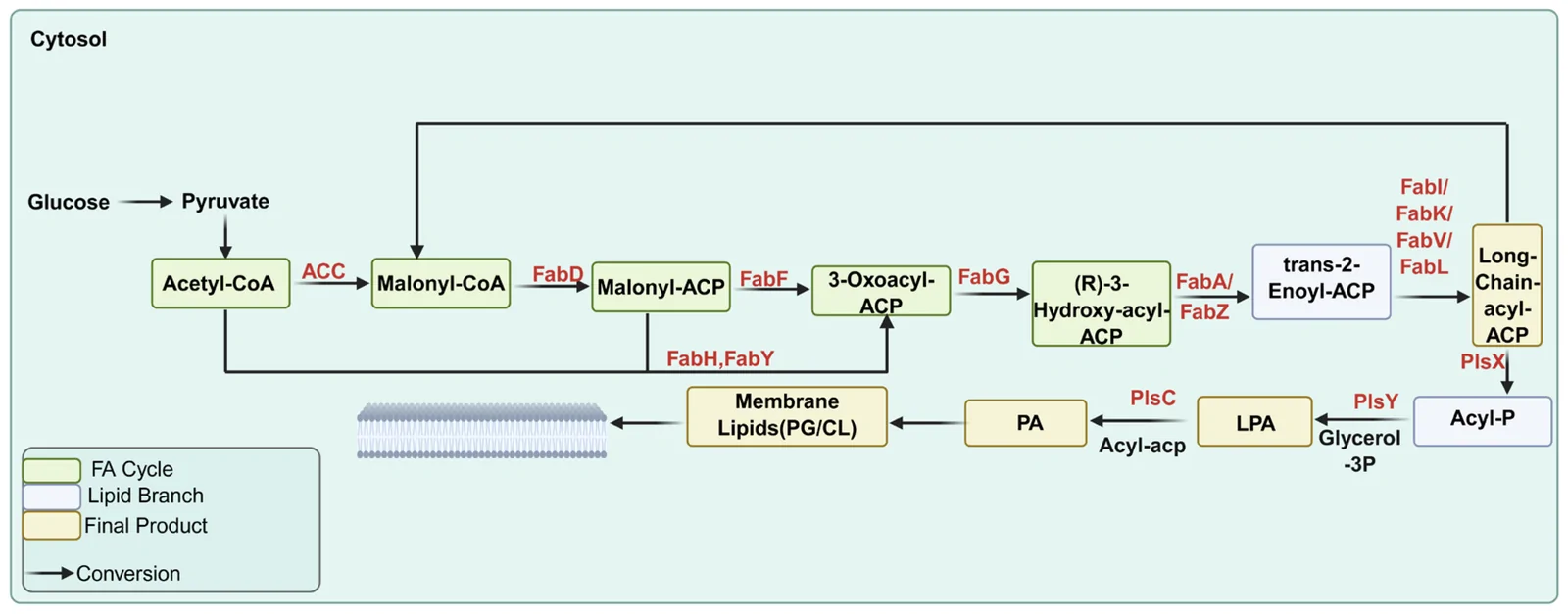
Lipid metabolic pathways.

Genome annotation showed that strains 31# and 7# had highly similar predicted lipid metabolism profiles (Figure 11). Both strains encoded the major components of the type II fatty acid synthase (FAS II) system, including FabD, FabH/FabF/FabY, FabG, FabA/FabZ, and FabI/FabK/FabV/FabL, as well as the PlsX/PlsY/PlsC system involved in membrane phospholipid biosynthesis. No clear strain-specific differences were identified in these core pathways, indicating broadly similar predicted capacities for fatty acid and membrane phospholipid biosynthesis.

## 4. Discussion

The present study identified two CLA-producing *L. plantarum* strains from Sichuan pickles and showed a clear strain-dependent difference after optimization. Strain 31# produced 66.50 ± 3.80 μg/mL total CLA in whey-based medium supplemented with soybean oil, whereas strain 7# produced 26.79 ± 1.09 μg/mL under its preferred mixed-oil condition. For comparison, *L. plantarum* lp15 isolated from naturally fermented Chinese pickles produced 26.1 μg/mL CLA [15], and *L. plantarum* Lp-01 reached 33.47 μg/mL under optimized pine nut oil fermentation conditions [20]. Higher values have also been reported, including 94.68 ± 3.57 μg/mL for an optimized *L. paracasei* strain [21] and a preliminary screening value of 95.25 μg/mL for *L. plantarum* [22]. Strain 31# therefore showed competitive CLA-producing capacity, although direct ranking across studies is limited by differences in substrates, culture conditions, extraction procedures, and analytical methods.

The difference between strains 31# and 7# became more apparent in whey-based media. In MRS containing free LA, the two strains produced similar amounts of CLA, but replacing MRS with whey-based medium increased CLA production much more strongly in strain 31#. Soybean oil further enhanced CLA production by strain 31#, whereas mixed oil produced only a modest improvement in strain 7#. Previous studies have likewise shown that medium composition and lipid substrate influence CLA formation by *L. plantarum* [14,20,22]. Oil-based substrates may alter the availability of free LA during fermentation, but lipase activity, free-LA release, residual LA, and conversion kinetics were not measured here. The mechanism underlying the apparent advantage of vegetable oils therefore remains unresolved.

The physiological results also distinguished the two strains. Both grew best at 30 °C and pH 6, and growth decreased as NaCl concentration increased. Strain 31# generally tolerated simulated gastrointestinal stresses better than strain 7#, particularly at pH 3.0. However, both strains survived poorly at pH 1.5, and strain 7# also declined during prolonged exposure to bile salts and simulated colonic fluid. Acid and bile tolerance are commonly used in preliminary screening of *L. plantarum* with probiotic potential [29], but the poor survival under severe gastric acidity is an unfavorable result. Thus, strain 31# showed better stress tolerance under several conditions, but neither strain displayed uniformly strong gastric resistance.

The probiotic-related assays showed complementary rather than uniformly superior traits. Strain 31# was more inhibitory toward *E. coli,* whereas strain 7# showed greater inhibition of *S. aureus* and higher cholesterol removal. Strain 31# also aggregated more rapidly and showed slightly greater hydrophobicity in some solvent systems. These assays are useful for initial in vitro screening of Lactobacillus isolates [36], but their interpretation is limited. Because untreated cell-free supernatants were used, the antimicrobial effect cannot be assigned specifically to bacteriocins, organic acids, or other metabolites. Likewise, cholesterol removal from broth does not establish an in vivo cholesterol-lowering effect, and autoaggregation or hydrophobicity does not demonstrate epithelial adhesion or intestinal colonization.

The safety profile was generally favorable, although the antibiotic susceptibility results require further evaluation. Neither strain showed detectable hemolysis, and both were susceptible to several antibiotics. Both strains were resistant to ciprofloxacin and trimethoprim-sulfamethoxazole; strain 31# was resistant to gentamicin, whereas strain 7# showed intermediate susceptibility, and strain 31# was also intermediately susceptible to penicillin. Reduced susceptibility to some antibiotics has been reported among Lactobacillus isolates [31,33]. However, disk-diffusion data alone cannot determine whether resistance is intrinsic, acquired, or potentially transferable. EFSA guidance recommends standardized assessment of bacterial susceptibility to antimicrobials of human and veterinary importance [35]. MIC determination and genome-based screening for acquired resistance determinants would strengthen the safety assessment.

The genomic comparison provides a useful framework for interpreting the phenotypic differences, but pathway annotation should not be equated with metabolic activity. The two strains had highly similar COG profiles, including the same number of genes assigned to lipid transport and metabolism, so broad functional-category counts do not explain their different CLA yields. A clearer genotype–phenotype correspondence was observed for galactose. Strain 31# fermented galactose and showed a more complete predicted galactose-utilization pathway, whereas strain 7# did not ferment galactose and lacked the annotated step linking galactose 1-phosphate to UDP-galactose. This supports a difference in genomic potential for galactose utilization, but not gene expression, pathway flux, ATP production, or a causal relationship with CLA formation. Both strains also fermented lactose despite incomplete predicted lactose-specific transport systems. In *L. lactis*, galactose uptake can occur through GalP or a galactose PTS [37], showing that alternative routes may operate when a canonical system appears incomplete.

A second genomic difference was identified in the acetate-forming branch. Both strains encoded LDH, Xfp, and enzymes associated with acetoin formation, whereas the PTA/ACK branch was complete in strain 31# and incomplete in strain 7#. In LAB, redistribution of pyruvate metabolism toward acetate can influence carbon and energy metabolism [38], making an ATP-related contribution of this branch a plausible hypothesis. The better survival of strain 31# at pH 3.0 is consistent with greater stress tolerance, but the present data do not establish a causal link among PTA/ACK activity, ATP generation, acid tolerance, and CLA production. Intracellular ATP, acetate flux, pathway expression, and enzyme activity were not measured. Studies in other microorganisms also show that pH regulation, organic acid metabolism, and environmental stress responses depend on functional and regulatory processes that cannot be inferred from pathway annotation alone [39,40,41]. Because these studies involve *B. subtilis*, *Schizosaccharomyces japonicus*, and *Streptomyces roseosporus*, respectively, they provide only indirect context for *L. plantarum*.

In contrast, the predicted lipid-metabolism pathways were highly similar between the two strains. Both encoded comparable FAS II components and the PlsX/PlsY/PlsC system, with no clear strain-specific differences. Bacterial membrane lipid remodeling is important for membrane homeostasis [42], but no membrane-lipid analysis was performed here, and the presence of PlsX/PlsY/PlsC does not demonstrate incorporation of CLA into membrane phospholipids. Short-chain alcohols have been reported to alter the fluidity of sarcoplasmic reticulum membranes [43], but this evidence comes from a nonbacterial membrane system and provides only indirect context. The candidate gene associated with linoleic acid isomerization was present in both genomes. Previous work established linoleic acid isomerase activity through heterologous expression and direct enzyme characterization [44], emphasizing that annotation alone is insufficient to confirm catalytic function.

Taken together, our genomic findings provide indirect, but not direct, support for the proposed link to CLA production. The differences observed in galactose utilization and the PTA/ACK acetate-forming branch may reflect broader differences in carbon and energy metabolism that could influence the cellular conditions supporting CLA bioconversion. However, these associations do not establish a direct mechanistic or causal relationship with CLA production. No strain-specific genomic determinant was identified that could directly explain the higher CLA yield of strain 31#. Therefore, the molecular basis for its higher CLA-producing capacity remains unresolved and requires functional validation.

Several limitations should be considered. Only two selected isolates underwent detailed physiological and genomic comparison, and the optimization experiments did not measure residual LA, substrate mass balance, or CLA formation kinetics. All probiotic-related assays were performed in vitro. More importantly, the genomic analyses describe predicted metabolic capacity rather than transcription, enzyme activity, or pathway flux. No transcriptomic, proteomic, targeted metabolomic, intracellular ATP, membrane-lipid, or enzyme-activity measurements were performed to validate the proposed links among carbohydrate utilization, acetate metabolism, stress tolerance, and CLA production. Future work should focus on CLA conversion kinetics, acetate formation, intracellular ATP, functional characterization of the candidate CLA-related enzyme, and expression of the galactose and PTA/ACK pathways. MIC-based susceptibility testing, genome-level resistance screening, direct epithelial-adhesion assays, and validation in food matrices will also be important.

Beyond the mechanistic implications, the present findings also have potential relevance for food and health-related applications. The CLA-producing capacity of strain 31#, particularly in a whey-based medium supplemented with an edible oil substrate, suggests its potential use in the development of CLA-enriched fermented foods. From an industrial perspective, the use of a whey-based fermentation medium may also provide an opportunity for the value-added utilization of whey-derived resources and may reduce dependence on more complex laboratory media. However, the economic feasibility of such an application was not evaluated in the present study and will require assessment at pilot and industrial scales. From a health-related perspective, microbial production of CLA may provide a food-based approach for increasing CLA levels in fermented products. Nevertheless, the present study did not evaluate CLA bioavailability, physiological or therapeutic effects, or in vivo probiotic efficacy. Therefore, these findings should be regarded as a basis for further product development rather than evidence of medical or clinical benefits.

## 5. Conclusions

This study identified two CLA-producing *Lactiplantibacillus plantarum* strains from Sichuan pickles, with strain 31# showing greater CLA-producing capacity and emerging as the more promising candidate. Phenotypic assays revealed strain-dependent differences in stress tolerance and probiotic-associated traits, while genomic analysis indicated differences in predicted carbohydrate and organic acid metabolism. The more complete predicted galactose-utilization and acetate-forming pathways in strain 31# may be associated with broader differences in metabolic performance, but they do not establish a direct mechanism for its higher CLA production. Overall, the genomic findings provide indirect support for the strain-dependent CLA phenotype, while the proposed association requires functional validation. However, these associations remain hypothetical because pathway annotation alone does not demonstrate gene expression, enzyme activity, metabolic flux, or causality. The preliminary safety and probiotic-related assays provide useful screening information but are not sufficient to establish probiotic efficacy or comprehensive safety for food use. Overall, strain 31# has potential for further development in CLA-enriched fermented foods. Future studies should functionally validate the proposed metabolic mechanisms and further assess antimicrobial susceptibility, adhesion, stability, and performance in relevant food matrices before firm mechanistic or probiotic claims are made.

## Figures and Tables

**Table 1:** Carbohydrate fermentation profiles of the strains.

Sugars	31#	7#
Glucose	+	+
Lactose	+	+
Galactose	+	−
Arabinose	−	−
Fructose	+	+
Xylose	−	−

Note: +, positive fermentation; −, negative fermentation.

**Table 2:** Biochemical characteristics of the strains.

Characteristics	31#	7#
Catalase	−	−
Litmus milk reduction	+	+
Litmus milk coagulation	+	+
Litmus milk peptonization	−	−
Stormy fermentation	−	−
Gelatin liquefaction	−	−
Indole test	−	−
H_2_S production	−	−
Motility	−	−
Methyl red test	−	−

Note: +, positive reaction; −, negative reaction.

**Table 3:** Antibiotic susceptibility of the two strains.

Characteristics	31#	7#
Erythromycin	S	S
Ciprofloxacin	R	R
Lincomycin	S	S
Cotrimoxazole	R	R
Chloramphenicol	S	S
Penicillin	S	I
Ampicillin	S	S
Ceftriaxone	S	S
Gentamicin	R	I
Tetracycline	S	S

Note: S, susceptible; I, intermediate; R, resistant.

**Table 4:** General genomic features of strains 31# and 7#.

Feature	31#	7#
Genome size (bp)	3266253	3264978
GC Content	44.54%	44.54%
CDS No.	3027	3024
rRNA No.	16	16
tRNA No.	65	65
CRISPR No.	2	2
Prophage No.	1	1
GI No.	5	6
IS No.	14	19

**Figure A1:**
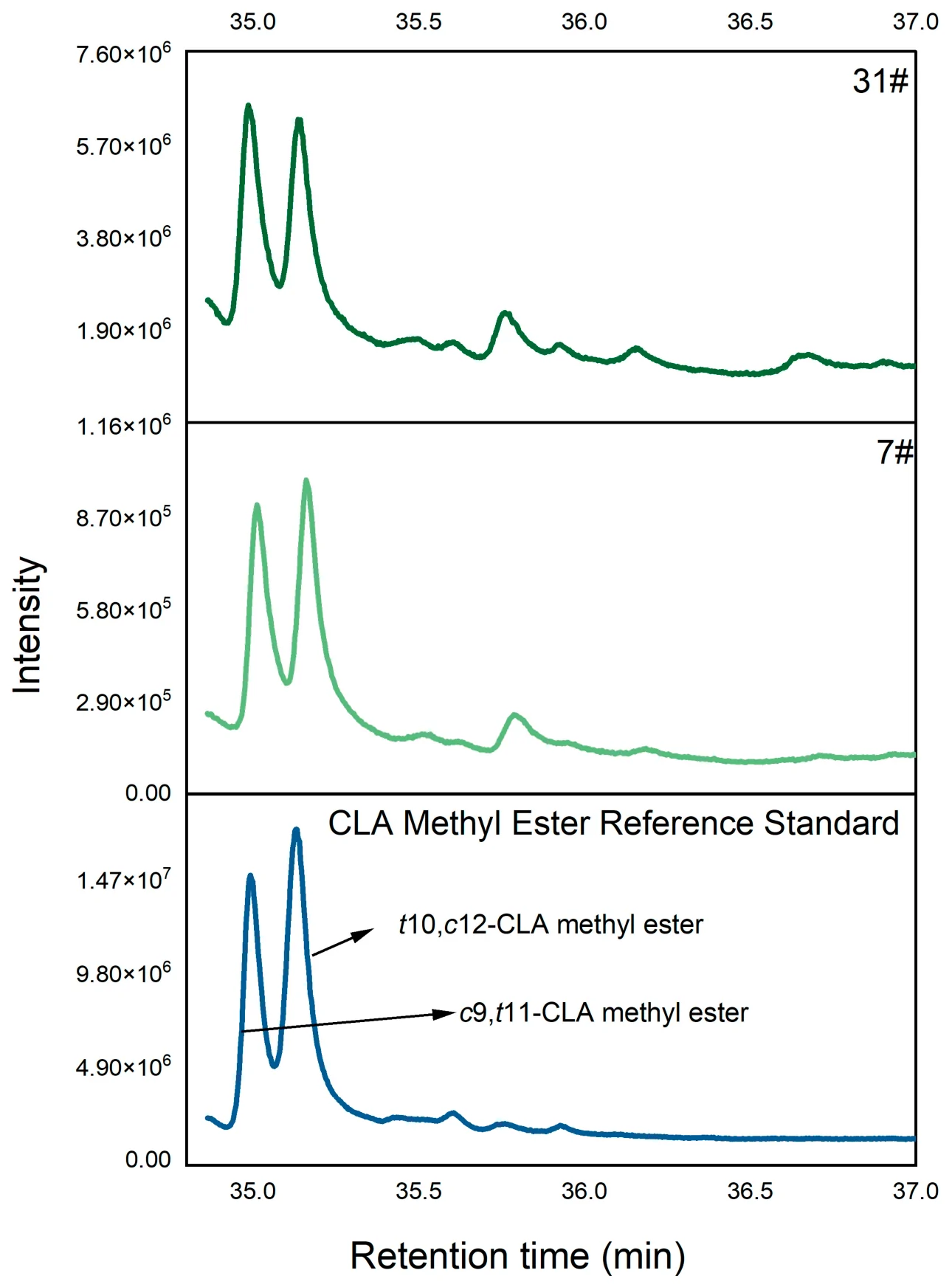
GC-Q-TOF chromatograms of the samples and the CLA methyl ester standard.

## Data Availability

The original contributions presented in this study are included in the article. Further inquiries can be directed to the corresponding authors.

## Abbreviations

The following abbreviations are used in this manuscript:

A_233_	Absorbance at 233 nm
ACC	Acetyl-CoA carboxylase
ACK	Acetate kinase
ACP	Acyl carrier protein
ANOVA	Analysis of variance
ATP	Adenosine triphosphate
BLAST	Basic Local Alignment Search Tool
CDS	Coding sequence
CFU	Colony-forming unit
CLA	Conjugated linoleic acid
COG	Clusters of Orthologous Groups
CoA	Coenzyme A
CRISPR	Clustered regularly interspaced short palindromic repeats
EFSA	European Food Safety Authority
EMP	Embden–Meyerhof–Parnas pathway
FAMEs	Fatty acid methyl esters
FAS	II type II fatty acid synthase
GC-Q-TOF	Gas chromatography–quadrupole time-of-flight mass spectrometry
GI	Genomic island
GRAS	Generally recognized as safe
HMS	Hexose monophosphate shunt
IS	Insertion sequence
KEGG	Kyoto Encyclopedia of Genes and Genomes
LA	Linoleic acid
LAB	Lactic acid bacteria
LDH	Lactate dehydrogenase
LPA	Lysophosphatidic acid
MEGA	Molecular Evolutionary Genetics Analysis
MLF	Malolactic fermentation
MRS	De Man, Rogosa and Sharpe
NAD^+^	Oxidized nicotinamide adenine dinucleotide
NADH	Reduced nicotinamide adenine dinucleotide
NJ	Neighbor-joining
OD_600_	Optical density at 600 nm
PA	Phosphatidic acid
PTA	Phosphotransacetylase
PTS	Phosphotransferase system
rRNA	Ribosomal RNA
SCF	Simulated colonic fluid
SD	Standard deviation
SGF	Simulated gastric fluid
SIF	Simulated intestinal fluid
tRNA	Transfer RNA
UV–Vis	Ultraviolet–visible
WGS	Whole-genome sequencing
Xfp	Phosphoketolase
XSO	*Xanthoceras sorbifolium* oil
Acetyl-P	Acetyl phosphate
Acyl-P	Acyl phosphate
AdhE	Bifunctional acetaldehyde-CoA/alcohol dehydrogenase
ALDC	α-acetolactate decarboxylase
ALS	Acetolactate synthase
AraA	L-arabinose isomerase
AraB	Ribulokinase
AraD	L-ribulose-5-phosphate 4-epimerase
CL	Cardiolipin
FA	Fatty acid
FabA	3-hydroxyacyl-ACP dehydratase/trans-2-decenoyl-ACP isomerase
FabD	ACP S-malonyltransferase
FabF	3-oxoacyl-ACP synthase II
FabG	3-oxoacyl-ACP reductase
FabH	3-oxoacyl-ACP synthase III
FabI	Enoyl-ACP reductase I
FabK	Enoyl-ACP reductase II
FabL	Enoyl-ACP reductase III
FabV	Enoyl-ACP reductase
FabY	Acetoacetyl-ACP synthase
FabZ	3-hydroxyacyl-ACP dehydratase
Fba	Fructose-bisphosphate aldolase
F1P	Fructose 1-phosphate
F6P	Fructose 6-phosphate
FruK	1-phosphofructokinase
G6P	Glucose 6-phosphate
GalK	Galactokinase
GalM	Aldose 1-epimerase (galactose mutarotase)
GalT	Galactose-1-phosphate uridylyltransferase
GAP	Glyceraldehyde 3-phosphate
Lac6P	Lactose 6-phosphate
LacA/LacB	Galactose-6-phosphate isomerase subunits
LacC	Tagatose-6-phosphate kinase
LacD	Tagatose-1,6-bisphosphate aldolase
LacG	6-phospho-β-galactosidase
Man6P	Mannose 6-phosphate
PDH	Pyruvate dehydrogenase
PEP	Phosphoenolpyruvate
PFL	Pyruvate formate-lyase
PG	Phosphatidylglycerol
Pgi	Glucose-6-phosphate isomerase
PfkA	6-phosphofructokinase
PlsC	1-acyl-sn-glycerol-3-phosphate acyltransferase
PlsX	Phosphate acyltransferase
PlsY	Acyl phosphate:glycerol-3-phosphate acyltransferase
PM	Permease-mediated transport
Pmi	Mannose-6-phosphate isomerase
Pyk	Pyruvate kinase
Pyr	Pyruvate
Sor6P	Sorbitol 6-phosphate
SrlD1/SrlD2	Sorbitol-6-phosphate dehydrogenases
XylA	Xylose isomerase
XylB	Xylulokinase

## Appendix A

The GC–Q-TOF chromatograms of the CLA methyl ester standards and the CLA methyl esters extracted from the fermentation broths are shown in Figure A1. Strain 31# was cultured in whey-based medium supplemented with soybean oil, whereas strain 7# was cultured in whey-based medium containing mixed oil. Under identical sample preparation and analytical conditions, two chromatographic peaks corresponding to *c*9,*t*11-CLA methyl ester and *t*10,*c*12-CLA methyl ester were detected in both fermented samples at retention times consistent with those of the standards. These results confirm that strains 31# and 7# are capable of converting LA into CLA.

## Appendix B

**Table A1:** Species identification and GenBank accession numbers of the bacterial strains.

Strain	Species	ID
1	*Levilactobacillus brevis*	PZ840564
2	*Lactiplantibacillus plantarum*	PZ840565
3	*Lactiplantibacillus plantarum*	PZ840566
5	*Lactiplantibacillus plantarum*	PZ840567
7	*Lactiplantibacillus plantarum*	PZ840568
8	*Lactiplantibacillus plantarum*	PZ840569
10	*Lactiplantibacillus plantarum*	PZ840570
11	*Lactiplantibacillus plantarum*	PZ840571
12	*Lactiplantibacillus plantarum*	PZ840572
13	*Lactiplantibacillus plantarum*	PZ840573
15	*Lactiplantibacillus plantarum*	PZ840574
16	*Levilactobacillus brevis*	PZ840575
17	*Levilactobacillus brevis*	PZ840576
18	*Lactiplantibacillus plantarum*	PZ840577
19	*Lactiplantibacillus plantarum*	PZ840578
20	*Levilactobacillus brevis*	PZ840579
21	*Lactiplantibacillus plantarum*	PZ840580
22	*Lactiplantibacillus plantarum*	PZ840581
23	*Lactiplantibacillus plantarum*	PZ840582
24	*Levilactobacillus brevis*	PZ840583
25	*Levilactobacillus brevis*	PZ840584
26	*Lactiplantibacillus plantarum*	PZ840585
27	*Lactiplantibacillus plantarum*	PZ840586
28	*Lactiplantibacillus plantarum*	PZ840587
30	*Levilactobacillus brevis*	PZ840588
31	*Lactiplantibacillus plantarum*	PZ840589
